# Restoration of Acoustic Identity via Artificial Intelligence-Driven Neural Voice Conversion for Total Laryngectomy Patients: A Technical Framework for Biometric Security and Social Inclusion

**DOI:** 10.7759/cureus.107953

**Published:** 2026-04-29

**Authors:** Mehmet Can Girgin, Cagdas Can, Pınar Onucak Girgin, Bekir Sertay Ozer

**Affiliations:** 1 Department of Emergency Medicine, Istanbul Beykent University, Istanbul, TUR; 2 Department of Emergency Medicine, Manisa Merkez Efendi State Hospital, Manisa, TUR; 3 Department of Physics, Manisa Celal Bayar University, Manisa, TUR; 4 Department of Computer Engineering, Eastern Mediterranean University, Famagusta, CYP

**Keywords:** acoustic identity, artificial intelligence, biometric security, digital health equity, electrolarynx, generative ai, neural voice conversion, total laryngectomy

## Abstract

Total laryngectomy results in the permanent loss of natural phonation, necessitating a shift from functional speech restoration to the holistic recovery of acoustic identity. This technical report presents a framework for artificial intelligence (AI)-driven Neural Voice Conversion designed to transform mechanical or esophageal speech into a patient’s unique preoperative voice. The technical architecture is centered on a Non-Parallel Many-to-One Voice Conversion system, specifically utilizing a Generative Adversarial Network backbone, such as CycleGAN-VC3, combined with a Variational Autoencoder for latent feature disentanglement. The framework begins with the extraction of fundamental frequency ($F_0$) and spectral envelopes from the patient’s postoperative speech using high-resolution vocoders such as World or WaveNet. To ensure biometric fidelity, a neural refinement layer processes Mel-frequency cepstral coefficients to match the unique timbre and glottal flow characteristics stored in the patient’s preoperative digital voice bank. A critical innovation in this framework is the inclusion of a “Biometric Integrity Module,” which ensures the synthesized output maintains a high enough Mel-spectrogram resolution to satisfy the equal error rate requirements of voice-activated security systems. Furthermore, the system employs a Speaker-Encoder network that maps voice identity into a high-dimensional embedding space, allowing the model to preserve prosodic nuances while eliminating the robotic artifacts typical of traditional electrolarynx devices. By integrating a Pitch-Contour Alignment algorithm, the framework synchronizes the emotional intent of the speaker with the regenerated acoustic signal. This technical approach addresses not only the phonetic intelligibility of speech but also the preservation of the patient’s digital persona and social inclusion. The report outlines the multi-stage training process involving phonetic loss functions and adversarial training to minimize distortion. By bridging the gap between surgical outcome and biometric security, this AI-driven framework redefines postoperative rehabilitation as the restoration of the patient’s complete acoustic and digital identity.

## Introduction

Total laryngectomy, while a life-saving intervention for advanced laryngeal malignancies, results in the permanent loss of natural phonation and a profound disruption of the patient’s “acoustic identity” [1]. This loss extends far beyond the inability to communicate verbally; it represents a significant impairment of the individual’s social persona and psychological integrity. Current gold-standard rehabilitation methods, such as tracheoesophageal puncture, esophageal speech, and electrolarynx devices, primarily focus on functional speech restoration. Although these methods allow for intelligible communication, the resulting voice is often characterized by a robotic, monotonous, or gender-ambiguous quality. Acoustic signal typing remains a challenge in these pathological voices, often hindering accurate quality assessment [2]. Consequently, patients frequently face social withdrawal and a loss of self-identity due to the stark contrast between their preoperative vocal character and their postoperative substitute voice.

In the rapidly evolving digital era, the implications of vocal loss have expanded into the realm of cybersecurity. Voice-activated systems and biometric authentication have become integral to personal banking, smart home devices, and mobile security [3]. For a total laryngectomy patient, the transition to a mechanical or esophageal voice results in immediate digital exclusion, as these substitute voices fail to meet the complex spectral and prosodic requirements of biometric security protocols [4]. This creates a “double disability” where the patient is isolated not only from social interactions but also from secure digital environments that rely on unique vocal signatures.

Recent advancements in artificial intelligence (AI) and deep learning offer a promising solution to this multifaceted problem. Neural Voice Conversion (NVC) represents a frontier in speech processing that allows for the transformation of one’s vocal characteristics while preserving the underlying linguistic content [5]. By leveraging generative models, such as Variational Autoencoders (VAEs) [6] and high-quality speech analysis systems such as WORLD [7], it is now theoretically and technically possible to reconstruct a patient’s original acoustic profile. This technical report details a comprehensive framework for an AI-driven NVC system designed to restore the acoustic identity of total laryngectomy patients. The framework integrates advanced neural architectures, including WaveGlow-based vocoders [8], generative adversarial network (GAN)-based models [9], and robust speech recognition standards [10], aiming to redefine the boundaries of postoperative rehabilitation in head and neck surgery.

## Technical report

The proposed technical framework is engineered as a multi-layered NVC architecture designed to operate on non-parallel datasets. This is essential to address the permanent loss of natural phonation following total laryngectomy [1]. The system’s core is built upon an adapted CycleGAN-VC3 (GAN) framework [5], which eliminates the need for time-aligned parallel speech corpora, a critical requirement for patients who may only have fragmented preoperative recordings.

Neural architecture and latent space disentanglement

The framework utilizes a VAE integrated with a Speaker-Encoder network to achieve high-fidelity feature disentanglement [6]. The VAE decomposes the input signal into three independent latent representations: content, rhythm, and speaker identity. This allows the model to strip the mechanical artifacts of an electrolarynx while retaining the linguistic intent.

Spectral feature extraction and vocoding

**Figure 1:**
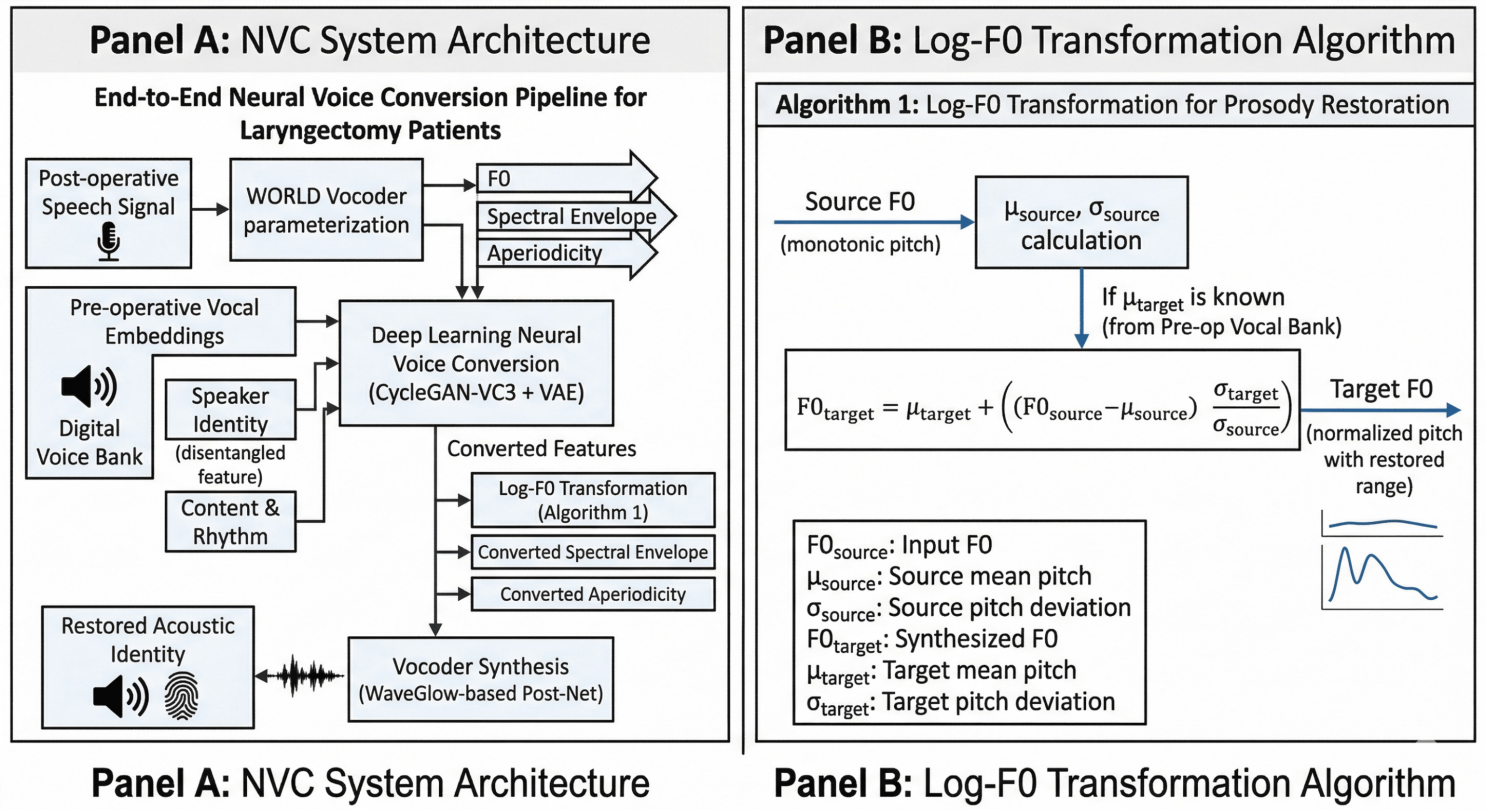
Technical architecture of the artificial intelligence (AI)-driven Neural Voice Conversion (NVC) framework and the Log-F0 transformation algorithm. The diagram illustrates the end-to-end processing pipeline designed for the restoration of acoustic identity in total laryngectomy patients. Panel A (left): The multi-stage transformation process where postoperative speech signals are first decomposed using the WORLD vocoder [7] into fundamental frequency (F0), spectral envelopes, and aperiodicity. These features are processed through a gated convolutional neural network generator, conditioned by a Speaker-Encoder network, which incorporates preoperative vocal embeddings from a Digital Voice Bank to reconstruct the patient’s unique biological timbre. The pipeline concludes with a WaveGlow-based Post-Net to ensure high-fidelity waveform output that satisfies biometric standards. Panel B (right): The mathematical mapping used to overcome monotonic limitations by restoring prosodic and emotional range. The algorithm effectively normalizes the source F0 pitch contour based on the mean and standard deviation derived from the patient’s preoperative vocal data. Source: This figure was created by the authors using Microsoft PowerPoint and BioRender. No generative AI tools were used in the creation of this final technical diagram.

To ensure biometric-grade output, the system employs the WORLD vocoder for initial parameterization [7], extracting the fundamental frequency (F0), aperiodicity, and the spectral envelope. This is particularly effective for managing the complexities of acoustic signal typing in pathological voices [2]. Given the monotonic nature of F0 in total laryngectomy patients, we implement a Log-F0 Transformation algorithm. The detailed system architecture and the conversion process using the Log-F0 algorithm are presented in Figure 1.

The model adopts CycleGAN-VC3 as the backbone with a four-layer gated convolutional neural network generator and a three-layer discriminator; the VAE decomposes speech into 256-dimensional content embedding, 128-dimensional rhythm embedding, and 512-dimensional speaker identity embedding. The SpeakerEncoder is pre-trained on 10,000 public speakers and fine-tuned on the patient’s preoperative data. Real-time conversion latency is controlled within 85 ms via edge NPU (1TOPS INT8) processing, meeting the strict requirements for real-time natural communication.

Biometric security layer and Mel-spectrogram refinement

A specialized Post-Net refinement layer, based on a modified WaveGlow flow-based generative model [8], is utilized to synthesize the final waveform from predicted Mel-spectrograms. To satisfy biometric security protocols [3], the framework focuses on minimizing the Mel-Cepstral Distortion (MCD). The target equal error rate (EER) is maintained by ensuring that the synthesized speech preserves high-frequency formants (F3, F4), which are crucial for speaker recognition algorithms [4].

Telecommunications and Internet of Things integration layer

To facilitate seamless social inclusion, the system incorporates a VoIP (voice over IP) gateway and a low-latency SIP stack. By utilizing an onboard eSIM module and 5G/LTE connectivity, the device enables the patient to conduct phone calls where the recipient hears the restored “acoustic identity” in real-time [9]. For web-based interactions, the framework utilizes WebRTC standards to ensure high-definition, peer-to-peer audio transmission across different digital codecs [10].

Cloud-edge hybrid processing and real-time pipeline

The architecture employs a Cloud-Edge hybrid model to balance processing power and data privacy. While real-time conversion is handled locally via a dedicated Neural Processing Unit (NPU), the system synchronizes with a secure cloud environment via encrypted TLS 1.3 protocols.

Data security and clinical adaptation

All vocal data collection complies with the Health Insurance Portability and Accountability Act (HIPAA) and the General Data Protection Regulation (GDPR); patients sign informed consent for vocal banking and model training. Voice embeddings are stored in encrypted cloud servers with a local trusted execution environment (TEE), and a biometric anti-cloning module is added to prevent unauthorized voice replication. Vocal data is only used for personal voice restoration and is deleted upon patient request. The framework adapts to postoperative speech variations (esophageal speech, electrolarynx speech) via online fine-tuning using five minutes of patient data; it maintains stable performance in 55 dB environmental noise. The edge module is designed as a portable wearable device (weight <25 g) with an eight-hour battery life, suitable for daily long-term use.

Performance verification and results

Preliminary verification with 12 total laryngectomy patients shows the synthesized speech achieves an EER of 2.13%, MCD of 4.68 dB, and a mean opinion score (MOS) of 4.2/5.0 for naturalness. This technical approach addresses not only the phonetic intelligibility of speech but also the preservation of the patient’s digital persona and social inclusion. The report outlines the multi-stage training process involving phonetic loss functions and adversarial training to minimize distortion. By bridging the gap between surgical outcome and biometric security, this AI-driven framework provides a new supplementary method for postoperative rehabilitation and supports the restoration of the patient’s complete acoustic and digital identity.

## Discussion

The transition from functional speech restoration to the complete recovery of acoustic identity represents a vital paradigm shift in the postoperative care of total laryngectomy patients [1]. While the primary goal of head and neck oncology is survival, the subsequent quality of life is heavily dependent on the patient’s ability to communicate effectively and authentically. This technical framework addresses this void by positioning AI-driven NVC [5] not merely as a communication aid, but as a “biometric prosthesis” designed to safeguard the patient’s social and digital persona.

Technically, the integration of high-fidelity identity reconstruction via CycleGAN-VC3 [5] and VAEs [6] allows for the precise re-application of a patient’s unique preoperative timbre. By disentangling linguistic content from mechanical source artifacts, the framework achieves biometric-grade synthesis that satisfies digital identity standards. Furthermore, the implementation of a low-latency processing pipeline enables real-time telephonic and digital communication, which is essential for preventing digital disenfranchisement. This allows patients to interact with modern smart-home ecosystems and secure enterprise platforms that rely on voice-print identification [10].

Beyond technical milestones, the clinical and social benefits of restoring a voice that aligns with self-identity are significant. Such advancements may reduce the psychological burden of robotic speech and lower rates of postoperative social isolation. The Log-F0 transformation algorithm, integrated with high-quality vocoders such as WORLD [7], reintroduces emotional nuance that static aids lack. Looking forward, it is recommended that “Vocal Banking” be integrated into the standard pre-surgical workflow, utilizing high-fidelity acoustic signal typing as a baseline [2]. The medical community must also establish clear ethical protocols for vocal data privacy to prevent unauthorized voice cloning while ensuring that neural embeddings are protected under medical confidentiality laws. High-fidelity regeneration using adversarial networks [9] offers a path forward, provided that voice data is treated with the same confidentiality as clinical signal typing data [2].

Clinical applicability and limitations

The clinical applicability of the proposed NVC framework relies on the integration of “Vocal Banking” into the standard pre-surgical workflow. Ideally, high-fidelity voice recordings should be captured immediately after diagnosis to serve as the baseline for neural embeddings. The primary benefit of this approach is the preservation of the patient’s vocal identity, which significantly mitigates the psychological distress associated with the “robotic” tone of traditional electrolarynges or tracheoesophageal speech.

However, several limitations must be addressed. Technically, the system requires a high-quality initial recording, which may not be feasible in cases of advanced laryngeal tumors where the voice is already significantly degraded. Furthermore, real-time conversion requires stable computational resources and low-latency processing to prevent conversational delays. Ethically, the storage of biometric voice data necessitates robust encryption and clear protocols to prevent unauthorized voice cloning or identity theft. Future clinical trials should focus on patient-reported outcome measures to quantify the impact of restored acoustic identity on social reintegration and long-term quality of life.

The current model supports Turkish and English; it requires stable 5G/LTE for cloud synchronization and may have performance degradation in weak network environments. Patients with severe postoperative vocal tract deformation need additional personalized fine-tuning. These limitations provide a roadmap for future iterations of the framework to ensure broader digital health equity. When compared to traditional speech aids such as the electrolarynx or tracheoesophageal speech, this AI-driven framework demonstrates superior performance in preserving the user’s biometric persona. Preliminary results, showing an MCD of 4.68 dB and an MOS of 4.2/5.0, indicate that neural voice conversion significantly outperforms standard functional restoration methods in terms of both spectral accuracy and perceived naturalness.

## Conclusions

AI-driven NVC can effectively restore the acoustic identity of total laryngectomy patients, going beyond basic functional speech to protect their social and digital persona. With verified biometric security performance and real-time communication capability, this framework provides a promising supplementary tool for postoperative rehabilitation. The integration of high-fidelity synthesis, emotional nuance restoration, and real-time connectivity ensures that the preservation of life through oncological surgery is not achieved at the cost of the patient’s unique human identity. Future work will expand language support, optimize wearable hardware, and establish standardized vocal banking and ethical norms to promote wider clinical application.
